# Cloning and characterization of filamentous temperature-sensitive protein Z from *Xanthomonas oryzae* pv*. Oryzae*

**DOI:** 10.1186/s40064-016-1876-3

**Published:** 2016-02-24

**Authors:** Leng Dai, Yunhong Huang, Yang Chen, Zhong-er Long

**Affiliations:** College of Life Science, Jiangxi Provincial Key Lab of Protection and Utilization of Subtropical Plant Resources, Jiangxi Normal University, Nanchang, 330022 Jiangxi China

**Keywords:** FtsZ, *Xanthomonas oryzae* pv*. Oryzae*, *Escherichia coli* BL21, Expression

## Abstract

The *ftsZ* gene from *Xanthomonas oryzae* pv*. Oryzae* was amplified by PCR with the specific primers, and the recombinant plasmid pET-22b-*ftsZ* was constructed successfully. The FtsZ with a 6× His tag was overexpressed in a soluble form in *Escherichia coli* BL21 and purified through a Ni-NTA agarose column. The purified recombinant FtsZ showed a single band on SDS-PAGE with an apparent molecular mass of about 44 kDa, and confirmed by western blotting analysis. The optimum temperature for GTPase activity of the recombined FtsZ was 50 °C, and the optimum pH was 7.0. The recombinant FtsZ showed good stability and retained >95 % activity at 50 °C for 240 min. The GTPase activity followed Michaelis–Menten kinetics with the K_M_ of 1.750 mM and the V_max_ of 0.155 nmol Pi/min/nmol FtsZ respectively.

## Background

The filamentous temperature-sensitive protein Z (FtsZ) plays an important role in the bacterial cell division. In the process of bacterial cell division, FtsZ forms single-stranded filaments and then the highly dynamic Z-ring scaffold, followed by the recruitment of other cell division proteins. Once the recruitment is accomplished, the filaments bend and Z ring contracts, leading to the closure of the septum and then the completion of the cell division, otherwise the bacterial cell division will be interrupted, which eventually result in bacterial apoptosis (Bi and Lutkenhaus 1991; Sossong et al. 1999). Due to its essential role and high conservation in bacteria, FtsZ is considered as an attractive target to develop the antibacterial agents with selective toxicity to bacterial pathogens (Margalit et al. 2004; Haydon et al. 2008; Kaur et al. 2010; Sass et al. 2011; Chan et al. 2013). Recently, a high-throughput screening model, based on inhibition of the GTPase activity of FtsZ, has been used to identify small molecules that target assembly-dependent GTPase activity of FtsZ, and then FtsZ inhibitors were developed as antibacterial agents inducing bacterial lethality (Chan et al. 2013). This has elicited much interest among researchers for the cloning of FtsZ from all kinds of bacteria (Thakur and Chakraborti 2006; Kiran et al. 2009; Modia et al. 2014); however, there is little known about its enzymatic characteristics.

*Xanthomonas oryzae* pv*. Oryzae* is a causal agent of bacterial blight which is one of the most widespread and devastating bacterial diseases of rice plants in China and many other rice-growing countries (González et al. 2012). The research showed that the bacterial blight can wither the rice leaf, interfere with the photosynthesis, increase the false grain, decrease the grain quality and ruin grain’s taste (Mew et al. 1993; Wu et al. 2013). Currently, the disease is managed by the use of resistant cultivars and antibacterial agents. However, the sensitivities of *X. oryzae* pv*. Oryzae* to the resistant cultivars and antibacterial agents have been found to decline, and resistant populations of *X. oryzae* pv*. Oryzae* have increased probably because of the wide use of the resistant cultivars and antibacterial agents, prompting a worldwide effort to develop new alternative antibacterial agents, especially with novel mechanisms of action (Davies 2011; Zhu et al. 2013).

Here we report the cloning and characterization of FtsZ from *X. oryzae* pv*. Oryzae*. The *ftsZ* gene from *X. oryzae* pv*. Oryzae* was cloned, and expressed in *Escherichia coli* BL21 (DE3) by IPTG induction. The purified FtsZ was analyzed by SDS-PAGE and corfirmed by western-blotting. The GTPase activity of FtsZ was determined by measuring the Pi released from GTP catalysied by FtsZ, and the enzymatic characteristics of FtsZ were investigated.

## Results

### Cloning of *ftsZ* gene

The genomic DNA of *X. oryzae* pv*. Oryzae* was extracted, and the concentration of DNA was detected by UV absorption at 280 and 260 nm by spectrophotometry (1100 UV/VIS, Shanghai). The density of genomic DNA was 81.8 μg/μl, the ratio of A_260nm_/A_280nm_ was 1.91, and then the genomic DNA was competent for PCR. The full length of an *ftsZ* gene was obtained by PCR using genomic DNA as a template. DNA sequencing showed that a 1261 bp DNA fragment containing the *ftsZ* gene was amplified by PCR with the gene specific primers, which exhibited highest similarity to the *ftsZ* gene (gene ID: 3264933) from *X. oryzae* pv. *Oryzae* KACC10331 with 100 % identity, indicating that the *ftsZ* gene had been obtained successfully and ligated into a pMD19-T vector to produce pMD19-*ftsZ*.

### Construction of recombinant expression vector

The recombinant expression vector pET-22b-*ftsZ* was transformed into *E. coli* BL21 (DE3). The positive clones were identified by plasmid PCR and restriction enzymes digestion. The sequencing results further showed that the pET-22b-*ftsZ* expression vector was successfully constructed.

### Expression and purification of the recombinant FtsZ

**Fig. 1:**
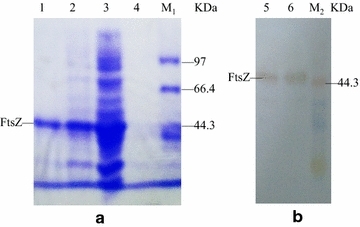
Analysis of the process for FtsZ expression and purification by SDS-PAGE (**a**) and western blotting analysis of the purified FtsZ (**b**). The FtsZ was expressed in *E. coli* BL21 (DE3)/pET22b-*ftsZ* induced by 0.6 mM IPTG at 37 °C for 3 h. Lane M_1_ and M_2_ protein Marker; *lane 1* purified FtsZ via Ni-NTA agarose column; *lane 2* supernatant after ultrasonication under induced conditions; *lane 3* total protein under induced conditions; *lane 4* supernatant of broth under induced conditions; *lane 5* supernatant of lysed *E. coli* BL21 cells containing pET-22b-*ftsZ*; *lane 6* purified FtsZ via Ni-NTA agarose column. The migration difference of the 44.3 kDa protein between (**a**) and (**b**) is due to gel electrophoresis with different pulse time

The recombinant vector pET-22b-*ftsZ* was transformed into *E. coli* BL21 (DE3), and expressed in the transformed cells upon induction with IPTG at 37 °C. The IPTG was used in the pET vector system because of the T7 promoter. According to the characteristics of the expression vector pET-22b (+) and the procedure for the construction of the expression vector, it can be predicted that there is a 6× His tag at the C-terminus of the expressed FtsZ (Termed the recombinant FtsZ), and the recombinant FtsZ can be purified by a Ni-NTA agarose column. The results of expression and purification of FtsZ were analyzed through SDS-PAGE (Fig. 1a). The FtsZ was expressed in *E. coli* BL21 (DE3)/pET22b- *ftsZ* induced by 0.6 mM IPTG at 37 °C, and determined in supernatant of lysed *E. coli* BL21 (DE3) cells containing pET-22b-*ftsZ*, which indicated that the fusion protein was produced in a soluble form. The SDS-PAGE showed that the FtsZ exhibited one specific band with a molecular mass of about 44 kDa, which is close to the theoretical value calculated from the deduced amino acid sequences of the *ftsZ* gene, indicating that it became electrophoretically pure. Meanwhile, the FtsZ was specifically recognized by the mouse anti-polyhistidine monoclonal antibody during the western blotting analysis (Fig. 1b), indicating that the FtsZ was indeed expressed in a recombinant protein with a 6× His tag.

### Bioinformatics of FtsZ

The coding nucleotide sequence of the *ftsZ* gene and the corresponding predicted amino acid sequences of FtsZ were analyzed. Nucleotide sequence analysis of the *ftsZ* gene revealed that the structural gene was composed of an open reading frame of 1242 bp. Analysis of the predicted protein sequence of FtsZ showed that it was composed of 422 amino acids with a predicted molecular mass of 43.967 kDa and an isoelectric point of 5.09. The expressed FtsZ was the monomeric protein with a 6× His tag and containing 34 basic amino acids and 49 acidic amino acids. The stability coefficient and hydrophic index of FtsZ was 34.26 and −0.06 respectively, that is to say that FtsZ belongs to a stabilizing and hydrophobic protein. A GTP binding site of GGGTGTG was seen in the recombinant FtsZ based on the primary structure with the Clustal software analysis, which indicated that the FtsZ from *X. oryzae* pv*. Oryzae* could have GTPase activity.

**Fig. 2:**
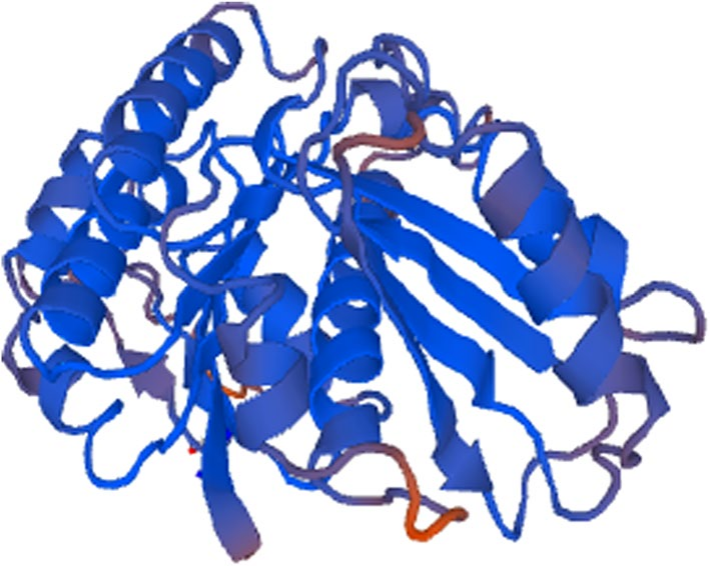
The proposed 3-D structural model of FtsZ. 3-D structural modelling of FtsZ was performed online with the Swiss-Model software

In addition, 3-D structural modelling of FtsZ was performed online with the Swiss-Model software (Fig. 2). The intricate spatial architecture showed that FtsZ contained 30.4 % α-helical structure, 11.8 % β-sheet structure and 57.8 % random coil structure, which was similar to FtsZ from other prokaryotes (Shin et al. 2013).

**Fig. 3:**
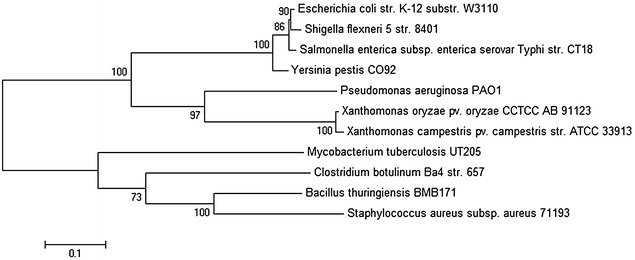
Phylogenetic analysis of FtsZ from *X. oryzae* pv*. Oryzae*. DNAMAN and MEGA5.1 software were used for phylogenetic analysis with the neighbour-joining method

Based on the deduced amino acid sequences of FtsZ from *X. oryzae* pv*. Oryzae* and other FtsZ, a neighbour-joining (NJ) tree was constructed, with the MEGA5.1 program, to investigate the evolutionary relationships (Fig. 3). The results revealed that FtsZ from *X. oryzae* pv*. Oryzae* was derived from the same ancestor as other FtsZ in microorganisms and had close relationship with the FtsZ from *X. campestris str.* ATCC 33913, which is consistent with the evolution of species.

### Enzymatic characteristics of the recombinant FtsZ

**Fig. 4:**
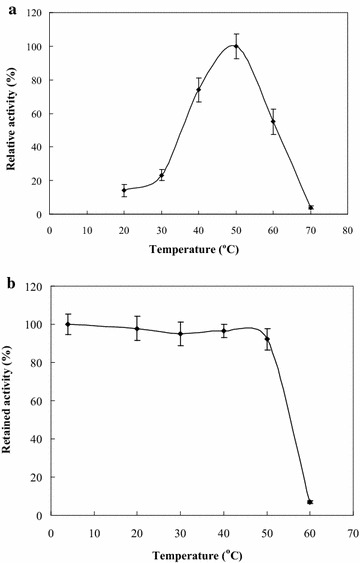
Effect of temperature on the GTPase activity (**a**) and the stability (**b**) of the recombinant FtsZ. The effect of temperature on GTPase activity of the recombinant FtsZ was determined by the GTPase activity assay with 5 mM GTP in buffer solution (pH 7.0) as substrate at temperature range of 20–70 °C, thermal stability of the recombinant FtsZ was determined by measuring the retained GTPase activity after incubation at 4–60 °C for 240 min. The relative GTPase activity of the recombinant FtsZ is the ratio of the GTPase activity at certain temperature and at optimum temperature (**a**), the retained GTPase activity of the recombinant FtsZ is the ratio of the GTPase activity after and before treatment (**b**)

The temperature had a remarkable effect on the GTPase activity and the stability of the recombinant FtsZ (Fig. 4). The optimum temperature for the GTPase activity of the recombinant FtsZ was about 50 °C. Meanwhile the recombinant FtsZ retained 100 % GTPase activity at 4 °C and >95 % at 50 °C for 240 min, whereas it rapidly lost its GTPase activity at 60 °C or higher.

**Fig. 5:**
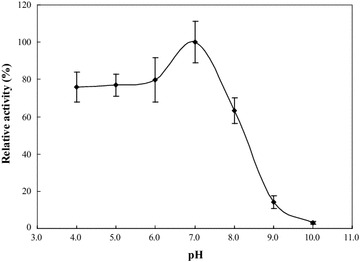
Effect of pH on the GTPase activity of the recombinant FtsZ. The effect of pH on GTPase activity of the recombinant FtsZ was determined by the GTPase activity assay with 5 mM GTP as substrate in the pH range of 4.0–10.0 at 50 °C. The relative GTPase activity of the recombinant FtsZ is the ratio of the GTPase activity at a certain pH and at optimum pH

The effect of pH on the GTPase activity of the recombinant FtsZ was shown in Fig. 5. It can be seen that the GTPase activity of the recombinant FtsZ increased with the increase of pH. The recombinant FtsZ had the best GTPase activity when the pH was 7.0, and quickly lost its activity at pH > 9.0. Therefore, pH 7.0 was selected as the optimum pH.

**Fig. 6:**
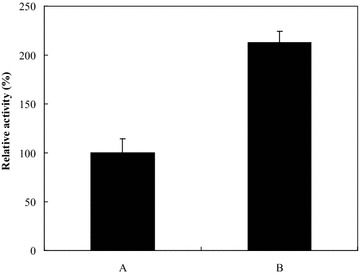
Effect of Mg^2+^ on the GTPase activity of the recombinant FtsZ. The effect of Mg^2+^ on GTPase activity of the recombinant FtsZ was determined by measuring the GTPase activity of FtsZ in the presence of Mg^2+^ at final concentration of 0 mM (**a**) and 5 mM (**b**) using the GTPase activity assay at 50 °C and pH 7.0. The relative GTPase activity of the recombinant FtsZ is the ratio of the GTPase activity in the presence and absence of Mg^2+^

Further, the effect of Mg^2+^ on GTPase activity of the recombinant FtsZ from *X. oryzae* pv*. Oryzae* was shown in Fig. 6. The GTPase activity of the recombinant FtsZ was increased by the addition of Mg^2+^. Thus, Mg^2+^ seems to be a co-factor of the intrinsic GTPase activity present in the recombinant FtsZ.

**Fig. 7:**
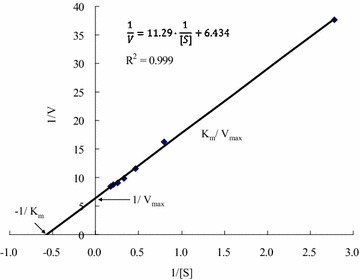
Analysis of the enzyme kinetic constants of the recombinant FtsZ. Michaelis–Menten kinetic experiments on GTPase activity of the recombinant FtsZ were performed with final concentration of 1–10 mM GTP as substrate at 50 °C and pH 7.0, and the kinetics K_M_ and V_max_ were calculated

The GTPase activity of the recombinant FtsZ was measured with an increasing concentration of GTP under the above optimal conditions. The kinetic constants were analysed by fitting to the Michaelis–Menten kinetics (Fig. 7) and it could be determined that the values of K_M_ and V_max_ were 1.68 mM and 0.155 nmol Pi/min/nmol of the recombinant FtsZ, respectively.

## Discussion

As it can be polymerized to form a Z ring acting as an essential skeleton during cell division, and is widely conserved in the bacterial kingdom, FtsZ is considered as an attractive target to develop the antibacterial agents with selective toxicity to bacterial pathogens and it has inspired many interests among researchers for the expression of FtsZ in vitro (Thakur and Chakraborti 2006; Kiran et al. 2009; Modia et al. 2014). According to the results of the literature survey, it is the first report on the expression in vitro of FtsZ from *X. oryzae* pv*. Oryzae* in this paper.

The dynamics of FtsZ polymerization depends on its properties as a GTPase. Therefore, the characteristics of the GTPase activity of FtsZ (in the form of the recombinant protein) were studied in this paper.

Firstly, based on the coding nucleotide sequence of the *ftsZ* gene and the corresponding predicted amino acid sequences, the structural characteristics of FtsZ from *X. oryzae* pv*. Oryzae* were analyzed by bioinformatics methods. The results showed that the structure of FtsZ from *X. oryzae* pv*. Oryzae* was similar to those from other bacteria, and the neighbour-joining tree showed that the phylogenetic relationships of FtsZ from different sources were consistent with the evolution of species (Fig. 5).

Secondly, the optimal conditions for the GTPase activity of FtsZ from *X. oryzae* pv*. Oryzae* were investigated, and the results showed that the optimum pH is 7.0 and the optimum temperature is about 50 °C. In addition, Mg^2+^ seems to be a co-factor of the intrinsic GTPase activity present in the recombinant FtsZ, which was similar to that of *Deinococcus radiodurans* (Modia et al. 2014).

Thirdly, based on the Michaelis–Menten equation, the values of K_M_ and V_max_ for the GTPase activity from *X. oryzae* pv*. Oryzae* FtsZ were 1.750 mM and 0.155 nmol Pi/min/nmol FtsZ, respectively. According to the results reported in the literature, the V_max_ of FtsZ from *X. oryzae* pv*. Oryzae* was lower than that of FtsZ from *E. coli* (19.758 nmol Pi/min/nmol FtsZ), from *Deinococcus radiodurans* (3.7 nmol Pi/min/nmol FtsZ), and from *Mycobacterium tuberculosis* (0.273 nmol Pi/min/nmol FtsZ) (Modia et al. 2014), perhaps the maximum reaction rate is related to the speed of cell division.

## Conclusions

The *ftsZ* gene from *X. oryzae* pv*. Oryzae* was cloned and overexpressed in *E. coli* BL21, the recombinant FtsZ was purified through a Ni-NTA agarose column, and we determined several biochemical properties of FtsZ, including the optimum parameters and kinetic constants of its intrinsic GTPase activity and structural protein features. The study may lay the foundation for further research and application of FtsZ from *X. oryzae* pv*. Oryzae*.

## Methods

### Bacterial strains, plasmids and reagents

*Xanthomonas oryzae* pv. *Oryzae* (CCTCC AB 91123) was obtained from China-Center for Type Culture Collection, Wuhan University (Wuhan, China), and cultured as previously described (Byul et al. 2011). Both *E. coli* DH5α used for gene cloning and *E. coli* BL21 (DE3) serving as an expression host were purchased from TaKaRa Biotechnology Co. Ltd (Dalian, China). *E. coli* strains were routinely grown in LB broth or on LB agar plates with 100 µg/ml of ampicillin at 37 °C. The pMD19-T vector and pET-22b (+) vector were purchased from Invitrogen Corp. (Shanghai, China), and used for cloning and expression studies, respectively.

DNA Marker, Protein Marker, GoldView, T4 DNA ligase, PrimeSTAR HS DNA Polymerase, QuickCutTM *Nde* I and QuickCutTM *Xho* I restriction enzymes were purchased from TaKaRa Biotechnology Co., Ltd (Dalian, China). A bacterial genome extraction kit, a DNA fragment purification kit, an agarose gel DNA purification kit and a MiniBEST Plasmid Purification Kit were purchased from Sangon Biotechnology Co., Ltd. (Shanghai, China). All aqueous solutions and buffers were prepared with water purified with an in-house Milli-Q Plus System (Millipore, Inc., Billerica, MA, USA). All the other chemicals were of analytical grade.

### Cloning of ftsZ gene

The genomic DNA of *X. oryzae* pv*. Oryzae* was extracted using the bacterial genome extraction kit according to the manual’s instructions. The *ftsZ* gene was amplified from the genomic DNA of *X. oryzae* pv*. Oryzae* by PCR with forward primer 5′-GGAATTCCATATGGCACATTTCGAACTGATTG-3′ and reverse primer 5′-CCGCTCGAGGTCGGCCTGGCGGCGCAGG-3′ (*Nde* I and *Xho* I restriction sites are underlined). The amplification conditions were as follows: denaturation for 1 min at 94 °C and then 30 cycles of 30 s at 94 °C, 30 s at 57 °C, and 90 s at 72 °C. The PCR product was verified by 1 % agarose gel electrophoresis and then extracted using the DNA fragment purification kit based on the manufacturer’s instructions. The product was cloned between the *Nde* I and *Xho* I sites of pMD19-T vector and confirmed by plasmid PCR and DNA sequencing.

### Construction of recombinant expression vector

The TA-cloned pMD19-*ftsZ* was digested by *Nde* I and *Xho* I restriction enzymes and followed ligation with the *Nde* I/*Xho* I digested pET-22b (+) to produce pET-22b-*ftsZ*. Finally, the recombinant plasmid was transformed into *E. coli* BL21 (DE3) with chemical transformation methods. The positive clones were identified by plasmid PCR and restriction enzymes digestion, and their DNA was verified by Sangon Biotechnology Co., Ltd (Shanghai, China).

### Expression and purification

The FtsZ was over expressed according to the methods described below. Recombinant *E. coli* BL21 (DE3) harboring the expression plasmid of pET-22b-*ftsZ* was pre-cultured in 5 ml LB broth medium with 100 µg/ml of ampicillin overnight at 37 °C on a rotary shaker at 200 r/min, and then inoculated at 2 % (v/v) into 250 ml flasks containing 100 ml LB broth medium supplemented with 100 µg/ml of ampicillin for soluble expression of protein. When the OD_600_ reached approximately 0.6, IPTG was added to 0.6 mM to induce protein expression for an additional 3 h at 37 °C on a rotary shaker at 200 r/min.

The FtsZ was purified according to the methods described below. Recombinant *E. coli* BL21 (DE3) cells were collected by centrifugation at 8000*g* for 5 min at 4 °C, suspended in lysis buffer (50 mM Tris–HCl, pH 8.0, with 100 mM NaCl and 200 µg/ml lysozyme) and lysed on ice by the sonication at 400 W for 99 cycles of 3 s working and 5 s intervals. The suspension was centrifuged at 12,000*g* for 20 min at 4 °C to remove the cell debris. The supernatant was collected and loaded onto the Ni-NTA agarose column (His tag affinity column) to purify recombinant protein according to the instructions of the manufacturer. After non-specifically bound proteins had been washed out, the FtsZ was eluted with one volume of elution buffer (50 mM Tris–HCl, pH 8.0, with 100 mM NaCl, and 500 mM imidazole). Finally, the FtsZ was dialyzed against buffer solution (50 mM Tris pH 8.0, 5 mM MgCl_2_ and 250 mM KCl) at 4 °C overnight and concentrated with polyethylene glycol 8000. The concentration of the purified FtsZ was determined by the Coomassie brilliant blue method with bovine serum albumin (BSA) as a standard and then stored at −20 °C for further study.

### SDS-PAGE

The protein samples were mixed with 4× SDS-PAGE loading buffer at the ratio 3:1 (v/v) and boiled for 5 min. The samples were run on 12 % SDS-PAGE gels at 100 V for 90 min with the Bio-Rad mini protein system (Bio-Rad Laboratories). The resolved protein samples were visualized by staining with Coomassie brilliant blue.

### Western blotting analysis

The presence of FtsZ in the purified fractions was confirmed by the western blotting analysis. For the western blotting, proteins were separated by SDS-PAGE on 12 % gel at 100 V for 60 min and transferred onto PVDF membrane (Bio-Rad) treated with methanol in transfer buffer (48 mM Tris, 39 mM glycine, 0.0375 % SDS, and 20 % (v/v) methanol) at room temperature, 67 mA for 1 h. Then the membrane was blocked with 5 % non-fat milk in PBS for 1 h at room temperature. After being washed twice with PBS Tween/Triton (20 mM Tris, 500 mM NaCl, 0.05 % Tween 20, and 0.2 % Triton X-100) and three times with PBS, the membrane was incubated for another hour with mouse anti-His antibody at a dilution of 1:5000 in PBS. After being washed four times with PBS, the membrane was incubated for 30 min with goat anti-mouse IgG antibody at a dilution of 1:5000 in PBS. After being washed four times with PBS, the membrane was taken into the DAB substrate solution. Then the target proteins were visualized with the enhanced chemiluminescence detection system.

### Bioinformatics analysis

The nucleotide and the predicted amino acid sequence were analyzed online (http://www.ncbi.nlm.nih.gov and http://www.expasy.org/proteomics), and the sequence comparison was conducted with the BLAST tool to find the homology of *X. oryzae* pv*. Oryzae* FtsZ with other FtsZ (http://www.ncbi.nlm.nih.gov). Swiss-Model analysis was also performed (http://swissmodel.expasy.org/). DNAMAN and MEGA5.1 software were used for sequence and phylogenetic analysis with the neighbour-joining method.

### GTPase activity assay

The GTPase activity of FtsZ was measured in a 96-well microplate with the malachite green method (Salvarelli et al. 2011). Reactions were carried out in 200 μl buffer solution (50 mM Tris, 5 mM MgCl_2_, 250 mM KCl, pH 7.5) with 5 mM GTP, and initiated by the addition of FtsZ at 37 °C. After 30 min, the reactions were quenched by adding 40 μl of above buffer solution containing EDTA (65 mM). The green malachite-molybdate reagent was added and absorbance at 620 nm was measured. A phosphate standard curve was done with Na_2_HPO_4_. The GTPase activity of FtsZ was determined from the slope of the linear part of the phosphate accumulation curve. One U of GTPase activity of FtsZ is defined as the amount of FtsZ required for the catalytic reaction to release 1 μmol Pi from GTP in 1 min. All experiments were performed in triplicate.

### Enzyme characterization

The optimum temperature for GTPase activity of FtsZ was determined by the GTPase activity assay with 5 mM GTP in buffer solution (pH7.0) as substrate at temperature range of 20–70 °C, thermal stability of FtsZ was determined by the retained GTPase activity after incubation at 4–60 °C for 240 min.

The optimum pH for GTPase activity of FtsZ was determined by the GTPase activity assay with 5 mM GTP as substrate in the pH range of 4.0–10.0 at optimum temperature.

The effect of Mg^2+^ on GTPase activity of the recombinant FtsZ was determined by measuring the GTPase activity of the recombinant FtsZ in the presence of Mg^2+^ at final concentration of 5 mM using the GTPase activity assay under the optimum temperature and pH.

Michaelis–Menten kinetic experiments on GTPase activity of FtsZ were performed with final concentration of 1–10 mM GTP as substrate at optimum temperature and pH, and the kinetics K_M_ and V_max_ were calculated.
